# Spatial variation in population-density of snow leopards in a multiple use landscape in Spiti Valley, Trans-Himalaya

**DOI:** 10.1371/journal.pone.0250900

**Published:** 2021-05-19

**Authors:** Rishi Kumar Sharma, Koustubh Sharma, David Borchers, Yash Veer Bhatnagar, Kulbhushansingh R. Suryawanshi, Charudutt Mishra

**Affiliations:** 1 Nature Conservation Foundation, Mysore, Karnataka, India; 2 Manipal University, Manipal, Karnataka, India; 3 Snow Leopard Trust, Seattle, WA, United States of America; 4 Snow Leopard Foundation in Kyrgyzstan, Bishkek, Kyrgyz Republic; 5 Centre for Research in Ecological and Environmental Monitoring, University of St. Andrews, St. Andrews, United Kingdom; U.S. Geological Survey, UNITED STATES

## Abstract

The endangered snow leopard Panthera uncia occurs in human use landscapes in the mountains of South and Central Asia. Conservationists generally agree that snow leopards must be conserved through a land-sharing approach, rather than land-sparing in the form of strictly protected areas. Effective conservation through land-sharing requires a good understanding of how snow leopards respond to human use of the landscape. Snow leopard density is expected to show spatial variation within a landscape because of variation in the intensity of human use and the quality of habitat. However, snow leopards have been difficult to enumerate and monitor. Variation in the density of snow leopards remains undocumented, and the impact of human use on their populations is poorly understood. We examined spatial variation in snow leopard density in Spiti Valley, an important snow leopard landscape in India, via spatially explicit capture-recapture analysis of camera trap data. We camera trapped an area encompassing a minimum convex polygon of 953 km2. Our best model estimated an overall density of 0.5 (95% CI: 0.31–0.82) mature snow leopards per 100 km2. Using AIC, our best model showed the density of snow leopards to depend on estimated wild prey density, movement about activity centres to depend on altitude, and the expected number of encounters at the activity centre to depend on topography. Models that also used livestock biomass as a density covariate ranked second, but the effect of livestock was weak. Our results highlight the importance of maintaining high density pockets of wild prey populations in multiple-use landscapes to enhance snow leopard conservation.

## Introduction

Large carnivores typically range over large areas [1], occur naturally at low densities [2] and exhibit elusive behaviour. Approximately 60% of the world’s largest carnivores are threatened with extinction [3]. Many large carnivore populations have undergone severe declines in their population size and distribution in the past few decades resulting in significant trophic cascades [4].

Evaluating the status of large carnivore species and the effectiveness of conservation actions requires rigorous monitoring of their populations. Inaccurate and imprecise estimates of population abundance can have larger cascading effects on the conservation of endangered species by their potential to influence a range of scientific inferences as well as conservation interventions. Large felids especially are typically solitary, secretive, and nocturnal, and live in densely vegetated habitats or remote regions, making it difficult to estimate their population- density.

The threatened snow leopard *Panthera uncia* is a typical example of a difficult to sample elusive carnivore that is reported to occur at relatively low population densities (0.15–3.88/100 km^2^) even in ideal habitats [5–7]. Snow leopards have relatively large home ranges, and of the 170 protected areas in the global snow leopard range, 40% are smaller than the home range size of a single adult male [8]. The distribution range of the snow leopard across high Mountains of Central and South high Mountains of Central and South Asia is comprised of multiple-use landscapes and is subject to pervasive human use, predominantly in the form of pastoralism and agro-pastoralism [9]. Over the past two decades, snow leopard habitats have also come under the increasing purview of developmental activities and mining [6], commercial livestock rearing such as cashmere goats [10], extraction of Cordyceps [11, 12], and tourism [13, 14].

Conservationists generally agree that snow leopards must be conserved amidst people, following a land-sharing approach, rather than overemphasising creation of strictly protected areas [8]. Such an approach, however, requires a good understanding of the impact of land use on snow leopard populations.

Within a landscape, snow leopard density can be expected to show spatial variation because of variation in the intensity of human use [14–16] and habitat quality (such as density and distribution of wild prey, topographical features and patterns of human development). A good understanding of such variation and its correlates is essential for designing appropriate, spatially explicit land-sharing strategies. However, snow leopard population abundances have been challenging to estimate, and spatial variation in their density remains undocumented and poorly understood. In this study, we assessed the spatial variation in snow leopard density and examined its ecological correlates in Spiti Valley, one of India’s most important snow leopard landscapes. We used the Akaike Information Criterion (AIC) to select spatial capture-recapture (SCR) models that explain spatial variation in density, encounter rate and habitat use.

## Materials and methods

### Study area

Spiti Valley (31⁰ 35’-33⁰ 0’N; 77⁰ 37’-78⁰35’E) is in the Indian state of Himachal Pradesh. Comprising of approximately 12,000 km^2^ of the catchment of the river Spiti, it is flanked by the Greater Himalaya in the south, Ladakh in the north and Tibet in the east. Lying in the Greater Himalaya’s rain-shadow, the region is cold and arid, with most of the precipitation in the form of snow. The primary vegetation type is dry alpine steppe and the region is characterised by the absence of trees. The landscape is rugged, and altitude ranges between 3000 to 6000 meters. Spiti experiences cold winters with the temperature dropping below -30⁰C, while summers have a mean maximum temperature of about 25⁰C. All necessary research permits were received before conducting the fieldwork from the Chief Wildlife Warden, Government of Himachal Pradesh, India.

**Fig 1:**
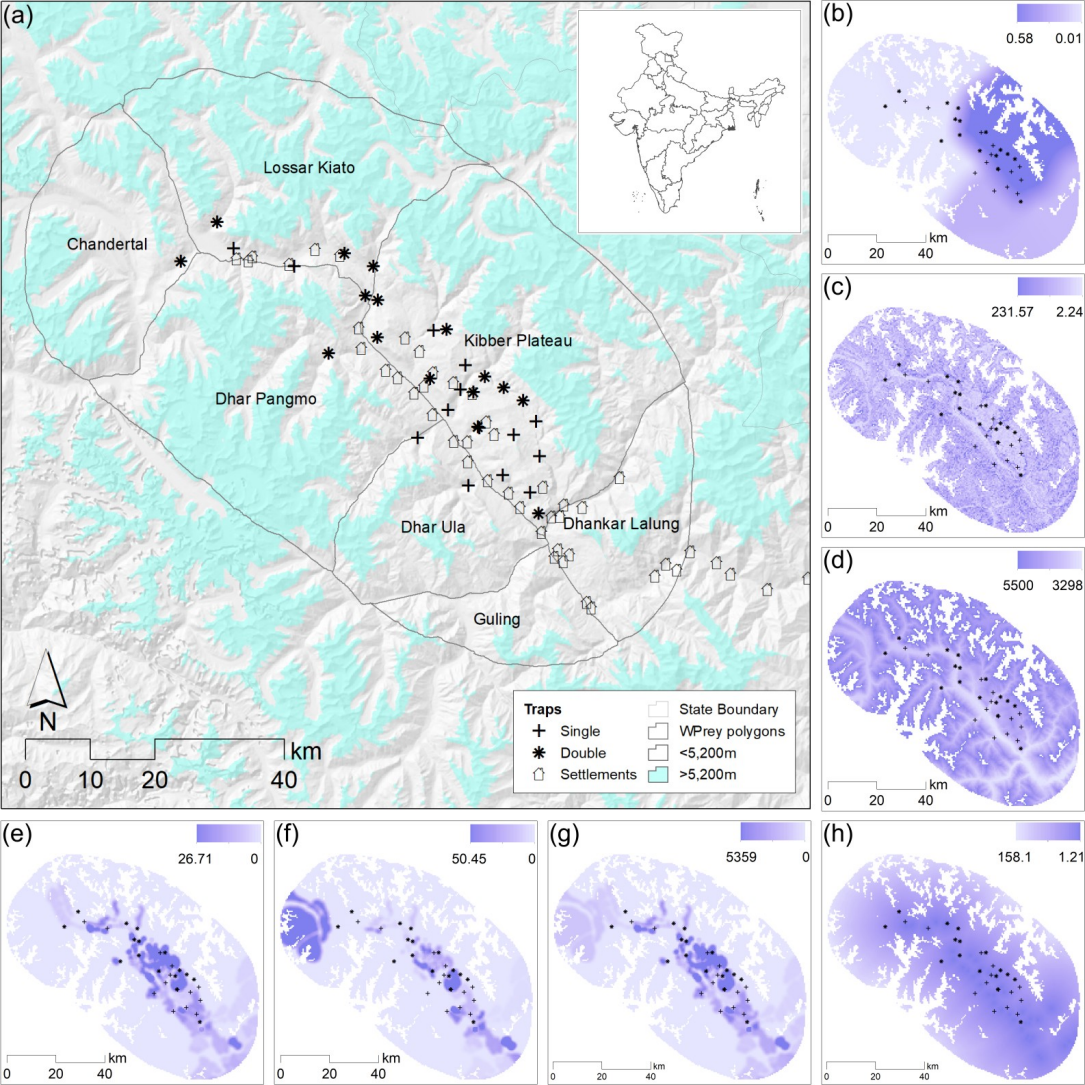
(a) Map of the study area showing camera trap locations and sampling region characterised by areas below 5200 meters. The inset map shows location of the study area in the state of Himachal Pradesh, India, and maps (b), (c), (d), (e), (f), (g) and (h) show spatial variation in wild prey density, ruggedness, altitude, density of large bodied livestock, density of small bodied livestock, overall livestock biomass, and least cost distance from settlements respectively within the area of integration. * The hill-shade, ruggedness and elevation data depicted the maps were developed by the authors using NASA Shuttle Radar Topography Mission (SRTM) Combined Image Data set 2014. Distributed by NASA EOSDIS Land Processes DAAC. https://doi.org/10.5067/MEaSUREs/SRTM/SRTMIMGM.003. Other spatial data were collected and prepared for visualization by the authors for the purpose of this manuscript.

In our study area (Fig 1), there were 50 hamlets and villages, with the number of houses ranging from 2 to 231 and their human populations ranging from 7 to 706. The human population density in the valley is < 2 persons per square kilometre. The local people are mainly agro-pastoralist, while transhumant pastoralists use parts of the valley in summers. Livestock species includes yak *Bos grunniens*, dzo (a male hybrid of cow and yak), dzomo (a female hybrid of cow and yak), cow *Bos indicus*, horse *Equus caballus*, goat *Carpa hircus*, sheep *Ovis aries* and donkey *E*. *asinus*. The key livestock grazing areas are located between 3,800 to 5,000 meters and communities have traditional grazing rights over rangelands. The region’s large mammalian fauna includes predators such as the snow leopard *Panthera uncia*, wolf *Canis lupus*, and wild ungulates such as ibex *Carpa sibirica* and bharal *Psedois nayaur*. Other mammalian species include hare *Lepus oiostolus*, red fox *Vulpes vulpes*, pale weasel *Mustela altaica*, stone marten *Martes foina* and pika *Ochotona spp*.

Snow leopards and wolves were historically persecuted in the region in retaliation for livestock depredation, though retaliatory killing has declined substantially owing to community-based conservation efforts.

### Estimating snow leopard population density using camera traps

We deployed Reconyx RM45 camera traps at 30 sites over an area of 953 km^2^ (Minimum Convex Polygon joining the outermost trap locations) with an average inter-trap distance of 4035 m (SE = 374m) (Fig 1). The camera traps were deployed from October 2011 to January 2012 for 80 days with an overall trap density of 3 camera traps per 100 km^2^ following recommendations of placing at least two traps per average home range [17] or at least two traps per average female home range [18]. The camera traps were deployed at sites where we encountered relatively high frequency of snow leopard signs such as scrapes, pugmarks, scats and scent marks, especially around terrain features that snow leopards are known to prefer for marking and movement such as ridgelines, cliffs and gully beds. We used a combination of single (n = 14) and double (n = 16) camera trap placement to optimise coverage and identification of individuals (Fig 1A). Double side camera traps were installed to enable capture of both side flanks of as many snow leopards as possible so they can be used to improve our ability to identify individuals with only single flank captures. Our cameras recorded snow leopards at 25 out of 30 sites without using any baits or scent lures.

Individual snow leopards captured in the images were identified based on their pelage patterns by two independent observers using at least three similarities or differences [5, 19]. There was no discrepancy in the identified individuals reported by the two observers. We only count each set of photographs as a new encounter when it was separated from another set of photographs from the same snow leopard by at least four hours. This was done to prevent the overdispersal of counts in using count detectors for our analysis. The mean time between consecutive encounters of the same animal on the same camera trap was 537 hours (95% CI: 409–665 hours) that ensures the validity of the count detector. We obtained a total of 2,830 snow leopard images from 124 encounters. A total of twelve encounters were discarded as the pictures were not good enough to identify the individuals. Using a mix of both side and right side only flanks, we obtained complete identification of 16 individual adult snow leopards. We assumed individuals moving about on their own (dispersed from their mother) to be mature individuals. There was no discrepancy between the two observers in individual identification of snow leopards. Following concerns raised by Johansson et al [20], we used the *Snow Leopard Identification*: *Training and Evaluation Toolkit* (https://camtraining.globalsnowleopard.org/leppe/login/) to test the skills of both of our observers in identifying snow leopards. Our observers scored 96.3% and 88.9% accuracy respectively in identifying snow leopards from 40 blind, independent trials, thus leaving us confident of identifying individuals with reasonable accuracy. Snow leopard capture histories were built using the standard count detector format of the `secr’ package in R [21] where each encounter of an identified cat was linked to a detector (camera trap site), whose location, period of operation and other relevant covariates were recorded in a separate table. We restricted the study period to 80 days and assumed that the population was closed and that there was no temporal effect on detection probability of snow leopards during the sampling period.

Typically, SCR models assume that the expected encounter rate depends on the Euclidean distance between detector and activity centre. This may not always be true in highly structured environments such as steep mountains. For example, we may record more encounters for a snow leopard in a distant trap than a closer trap if the habitat between the closer trap and activity centre has a higher resistance to movement (e.g. a deep gorge separating two detector locations). Royle *et al*. [22] and Sutherland *et al*. [23] proposed replacing Euclidian distance with a least-cost path distance (ecological distance) in which movement cost depends on the habitat. The method involves the estimation of movement cost parameter(s) simultaneously with other SCR parameters. Sutherland et al. [23] demonstrated that violations of the Euclidean distance assumption could bias estimates of density, and they suggest that least-cost distance be tested in highly structured landscapes.

We used the maximum likelihood-based SCR models [24] to estimate density while investigating the effect of least-cost path distance on movement, using package ‘secr’ [21] in R [25]. The method involves integration over a 2-dimensional region containing the possible (and unknown) locations of the activity centres of animals at risk of detection. The region of integration is based on a polygon extending a certain distance (the buffer width) beyond the outermost traps.

We used the inbuilt ‘suggest buffer’ function of ‘secr’ to arrive at a buffer of 24,000 meters assuming it to be wide enough to keep any bias in estimated densities as acceptably small (i.e. snow leopards with activity centres beyond 24 km from the outermost traps had a negligible probability of being captured in the detectors). Areas above 5,200 m from mean sea level were excluded from the set of possible activity center locations because they are mostly devoid of vegetation and prey species. We defined an integration area with a spacing of 500 m, resulting in 20,513 pixels for the entire integration area. We used the model with minimum AIC to estimate population size (*N*) and density (*D*) over the integration region [26], but use a model averaged density surface to present the distribution of the density of snow leopard activity centres.

*Spatial capture-recapture models* The spatial distribution model in SCR is a spatial Poisson process for animal activity centres whose intensity (expected number of animal activity centres per unit area) can be homogeneous (constant over space) or inhomogeneous (varying over space) [24]. We use the notation *D(x;****θ****)* for density, signifying that density is a function of activity centre location, *x*, which is a vector representing the x and y coordinates of an activity centre, and of parameters represented by the vector ***θ***.

We fitted SCR models with various combinations of covariates defined a priori. A candidate model set was developed to investigate the effects of various covariates potentially influencing snow leopard behaviour, ecology and natural history. We investigated models with various combinations of covariates for the density model, the encounter function intercept model, and the encounter function range model. The general forms of the density model, and encounter function intercept and range models, respectively, are as follows:

$$log\{D(s)\}=\theta_{0}+\sum_{d}\theta_{d}x_{d}(s)$$ (1)

$$log\{\lambda_{0}\}=\phi_{0}+\sum_{l}\phi_{l}x_{l}$$ (2)

$$log\{\sigma \}=\beta_{0}+\sum_{i}\beta_{i}x_{i}$$ (3)

where

*x*_*d*_(*s*) is the *d*th spatially referenced covariate at location ***s*** that affects density (*D*), and *θ*_0_ and *θ*_*d*_ are the density intercept parameter and *d*th regression parameter;
*x*_*l*_ is the *l*th covariate that affects expected encounter rate at distance zero (*λ*_0_), and *ϕ*_0_ and *ϕ*_*l*_ are the intercept parameter and *l*th regression parameter for expected encounter rate at distance zero;
*x*_*i*_ is the *i*th covariate that affects the encounter rate range parameter (*σ*), and *β*_0_ and *β*_*i*_ are the range intercept parameter and *i*th regression parameter.

Half-normal encounter function forms were used, such that the expected number of encounters of an animal at a camera that is a distance *d* from its activity centre is *E*(*n*) = *λ*_0_*exp*{−*d*^2^2*σ*^2^}.

For snow leopard density, we considered models in which the *x*_*d*_(*s*)s were wild prey density, large and small livestock density, overall livestock biomass, least cost distance from settlement, terrain ruggedness and altitude at *s*. We investigated the effect of topographies (a factor with levels “ridgeline”, “cliff” or “gully bed”), and the effect of single and double camera traps on the encounter function intercept and range parameters. We also investigated models in which movement cost depends on altitude by replacing Euclidian distance with a least-cost path distance in which movement cost depended on altitude.

We modelled *D*(*s*) as a function of six spatial covariates (*x*_*d*_(*s*)s) that could affect snow leopard density (Fig 1). These included terrain ruggedness (typical snow leopard habitats are steep and rugged) [27], altitude (snow leopard densities are known to be a function of altitude) [28], wild prey density (believed to be the main determinant of snow leopard population abundance) [29], stocking density of large-bodied livestock and small-bodied livestock (potential prey for snow leopards, source of disturbance, and competitors for wild prey) [27–30]. The terrain ruggedness across the integration region ranged from 12.63 to 74.55 (Mean = 40.97, SD = 9.45), the altitude ranged from 3298 meters to 5500 meters above mean sea level (Mean = 4775, SD = 509), the least cost distance (after estimating the associated parameter) from nearest village, using elevation as a cost function from 1.21 to 1580.82 (Mean = 599.81, SD = 415.62). The density of large livestock ranged from 0 to 14.11 per km^2^ (Mean = 1.41, SD = 2.21) while that of the small bodied livestock ranged between 0 to 11.39 (Mean = 0.89, SD = 1.75) per km^2^. The mean livestock biomass ranged between 0 and 5,539 kg (Mean = 230.71, SD = 471.06).

Terrain ruggedness was derived using the terrain ruggedness index [31] from a 30 × 30 meter Digital Elevation Model from Aster Global Digital Elevation Model data using the terrain analysis plugin in the Quantum GIS 3.16.3 software [32]. Livestock density was determined through a door to door censuses in 51 villages in the integration region. The pastures used by each village were mapped using Google Earth and livestock stocking densities for small- and large-bodied livestock were computed separately (as they are often herded separately [33] by dividing the total livestock heads using a pasture, by the area of the pasture in square kilometres. We used the average biomass of large-bodied and small-bodied livestock [34] to estimate the livestock biomass availability to snow leopards across the integration region. We smoothened the livestock density and ruggedness surfaces across the integration region by averaging over a moving window of 1.5km.

The abundance of wild prey, which primarily included blue sheep and ibex, was estimated using the double observer survey technique for the entire integration region [35] (Table 1) between April and June 2012. Four teams, comprising two observers each, carried out the surveys for eight days to cover the entire study area. Observers recorded the GPS coordinates of the sightings, the group size and age-sex classification of the groups encountered. The unique identity of each observed ungulate group was established through immediate post survey discussions between two observers using the age-sex classification, size and the location information of sightings [35]. The integration region was divided into seven blocks delineated based on natural topographic features in the landscape such as rivers and contours of prominent ridgelines. For each block, the cumulative number of wild ungulates encountered by the two observers were calculated. The relative density of wild ungulates for each block was estimated as total number of wild prey in a block divided by the size of the survey block (Table 2). The wild prey density surface was smoothened by averaging over a moving window of 5 km.

**Table 1:** Estimates of abundance of blue sheep and ibex using double observer approach in Spiti valley.

Variable	Blue sheep	Ibex	Overall (Blue sheep & Ibex)
*C*	75	18	93
S_1_	18	5	23
S_2_	6	1	7
$\hat{G}$	100.42	24.26	124.71
*SE $\left(\hat{G}\right)$*	3.45	0.57	1.51
$\hat{U}$	14.85	12.38	14.37
*SE)*	1.25	1.48	1.04
N	1470	297	1767
$\hat{N}$	1491	300	1792
*SE* $\left(\hat{N}\right)$	126.76	36.59	132.22
± 95% Confidence Interval	1239-1743-	227–373	1529–2055
P_1_	0.92	0.94	0.93
P_2_	0.80	0.78	0.80

*C* is the number of groups seen in both surveys; S_1_ is the number of groups seen in first survey only; S_2_ is the number of groups seen in second survey only; $\hat{G}$ is the estimated number of groups; N is the naïve population estimate; $\hat{N}$ is the estimated population size; P_1_ and P_2_ are the means of the estimated detection probability for observers one and two, respectively.

**Table 2:** The seven regions of Spiti Valley showing the estimated wild ungulate abundance across different regions.

Region	Area (km^2^)	Wild Prey Density (per km^2^)	LCL	UCL
**Chandertal**	768	0.01	0.07	0.70
**Kibber Plateau**	1623	0.58	0.56	2.04
**Dhar Ula**	578	0.29	0.26	0.87
**Dhankar Lalung**	513	0.23	0.20	0.81
**Dhar Pangmo**	1423	0.01	0.07	0.71
**Guiling**	346	0.25	0.22	0.83
**Lossar Kiato**	1117	0.04	0.08	0.71

LCL is the 95% interval lower limit, UCL is the 95% confidence interval upper limit.

We developed an *a priori* set of models that we anticipated would best explain the variation in the density of snow leopards. Our global (most complex) model included terrain ruggedness, linear and quadratic effect of altitude above mean sea level, density of wild prey, stocking density of small-bodied livestock, stocking density of large-bodied livestock, least-cost distance from settlements considering altitudinal gain as the added cost, and cumulative livestock biomass. We then fitted 20 candidate sub-models using subsets of the variables used in the global model. Each candidate sub-model represented a specific hypothesis about the relationship between snow leopard density and how snow leopards use space about their activity centres, and explanatory variables. We used Akaike’s Information Criterion (AIC) for model selection [36]. All data analysis was implemented using package secr [21] in program R [25].

## Results

The double observer surveys for wild prey yielded abundances of 300±72 (95% CI) ibex and 1491± 251 blue sheep in the entire study area (Table 1). The wild prey densities within the survey blocks ranged from 0.01 to 0.58 per km^2^ (Table 2).

**Fig 2:**
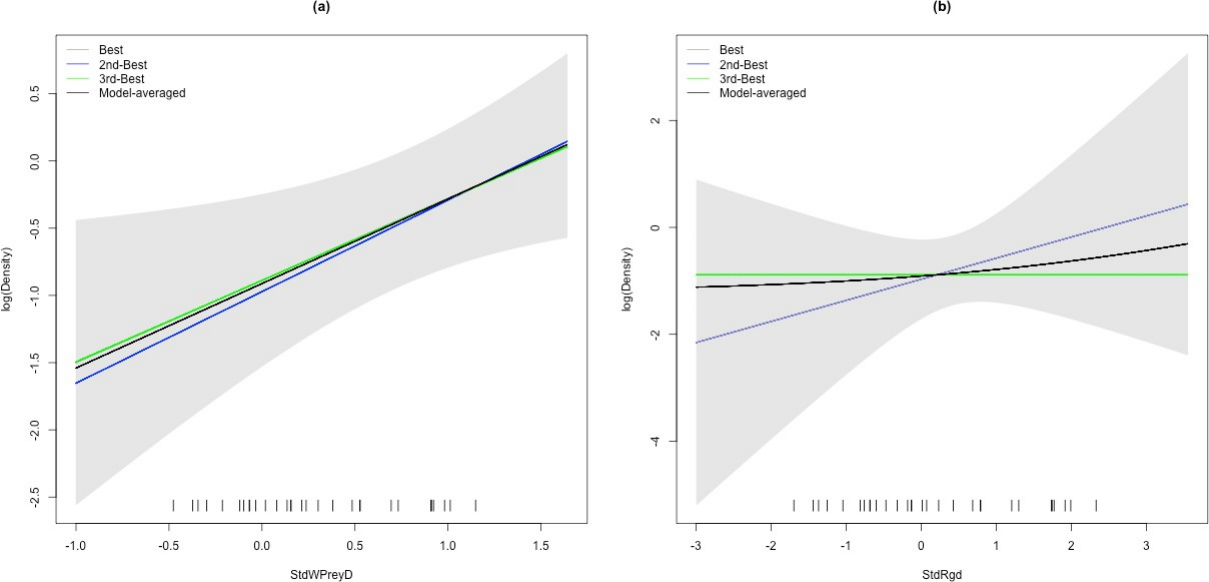
Model averaged snow leopard density estimated using the top three models as a function of (a) wild prey density (StdWPreyD) and (b) ruggedness (StdRgd) in Spiti Valley, India. The tick-marks on the x-axis show the placement of camera traps in the StdWPreyD and StdRgd dimension, with the range of the x axis indicating the range of StdWPreyD and StdRgd values in the data.

We obtained 112 captures of 16 individual snow leopards over a sampling period of 80 days. Snow leopards were captured at 25 of the 30 camera trap sites. The estimate of snow leopard abundance from the top model with the minimum AIC was 26 (95% CI: 16–42) for an area of 5,144 km^2^ covering the area of integration (integration region), resulting in an average density of 0.5 (SE = 0.13, 95% CI: 0.31–0.82) per 100 km^2^. Our camera traps spanned the covariate space of the wild prey density reasonably well (Fig 2).

Given the small difference in the AIC values of the top models, we model-averaged the density surface across the integration region using the top five models within five delta AICs from the top model whose cumulative AIC weight was 0.88. The estimates from the model averaged surface ranged from 0.16 to 2.08 per 100 km^2^ across the region of integration (Table 2). All top models predicted snow leopard habitat use around their activity centres to be a function of altitude: The conductance coefficient associated with least cost distance in the best model (parameter *α*_2_ of [23, 37] was estimated to be 0.36 (SE = 0.08), indicating that relatively higher altitudes within the study area boundaries were more conducive to snow leopard movement. Similarly, all top models used number of cameras per station (AIC weight = 1.0) and topography (AIC weight = 0.96) as covariates affecting encounter rate at an activity centre. The models using wild prey density as a covariate affecting snow leopard density had a cumulative AIC weight of 0.88, followed by ruggedness (AIC weight = 0.33) and then other covariates (Table 3). The coefficients for the top covariates were consistently significant at the 5% level (Table 4). The top 3 models with delta AIC less than 2 indicated that wild prey density and ruggedness positively affected snow leopard density across the landscape. The expected encounter rate for a trap with two cameras at the activity centre was consistently higher than the one with a single camera. Additionally, camera traps in gully beds and ridgelines had a higher expected encounter rate for cameras at the activity centre.

**Table 3:** Top eight models with cumulative Akaike’s Information Criterion (AIC) weight of 0.95, ranked on the basis of AIC for spatial capture recapture estimates of snow leopard density in a multiple use landscape.

Model	npar	logLik	AIC	dAIC	AICwt
**D~WildPrey; lambda0~Topo + Cameras; sigma~1 noneuc~Altitude**	8	-150.85	317.702	0	0.3717
**D~Ruggedness + WildPrey; lambda0~Topo + Cameras; sigma~1; noneuc~Altitude**	9	-150.43	318.858	1.156	0.2086
**D~WildPrey; lambda0~Cameras; sigma~1; noneuc~Altitude**	6	-153.79	319.587	1.885	0.1449
**D~Ruggedness + WildPrey; lambda0~Cameras; sigma~1; noneuc~Altitude**	7	-153.06	320.11	2.408	0.1115
**D~WildPrey’ lambda0~Topo; sigma~1; noneuc~Altitude**	7	-154.09	322.184	4.482	0.0395
**D~1; lambda0~Cameras; sigma~1 noneuc~Altitude**	5	-156.51	323.027	5.325	0.0259
**D~Altitude; lambda0~Cameras; sigma~1; noneuc~Altitude**	6	-155.57	323.137	5.435	0.0245
**D~Altitude + Altitude^2; lambda0~Cameras; sigma~1; noneuc~Altitude**	7	-154.85	323.707	6.005	0.0185

Spatial capture recapture models are described using the following notation: “~1” indicates that the RHS of Eqs (1) to (3) contains only an intercept term; “~x” means that it contains an intercept and covariate “x”; “~x+y” means that it contains an intercept and covariates “x” and “y”; “x*y” indicates that it contains the x and y main effects and an interaction between x and y, npar is number of parameters in the model, and logLik is the maximum log-likelihood. The difference between the AIC and the minimum AIC for the given candidate model set is denoted by dAIC, while the associated weight is denoted by AICwt.

D~, lambda0~, sigma~ and noneuc~ represent the density model, the encounter function intercept model, the encounter function range model and the conductance model respectively, modelled as functions of covariates or only a constant term.

Ruggedness is the terrain ruggedness index, Altitude is elevation above mean sea level, WildPrey is density of wild prey, LargeLS is stocking density of large bodies livestock, SmallLS is stocking density of small bodied livestock, and LSBiomass is the total livestock biomass. Cameras is the binary trap covariate indicating whether or not two cameras were deployed at a camera trap, and Topo is the factor variable indicating the placement of a camera trap at a ridgeline, cliff or gully bed.

**Table 4:** Coefficients of covariates from the top five models depicting their relative importance and corresponding effect on the model.

Estimate	Covariate	Mean covariate value before transformation (SE: Standard Error)	Cumulative AIC Weight	Coefficient β (95% CI)				
				Model 1	Model 2	Model 3	Model 4	Model 5
**AIC Weight**		**NA**	**NA**	0.37	0.21	0.15	0.11	0.04
**Density**	Intercept	NA	NA	-10.1 (-10.74 - -9.46)	-10.18 (10.93–9.44)	-10.1 (-10.74 - -9.46)	-10.23 (-11.03 - -9.44)	-10.10 (-10.70 - -9.51)
	Wild Prey Density	0.23 (0.22)	0.88	0.61 (0.08–1.14)	0.68 (0.13–1.24)	0.61 (0.07–1.14)	0.69 (0.14–1.24)	0.59 (0.08–1.10)
	Ruggedness	40.97 (9.45)	0.33	-	0.40 (-0.48–1.27)	-	0.51 (-0.35–1.38)	-
	Altitude	4775 (509)	0.04	-		-	-	-
	Altitude^2^	NA	0.02	-		-	-	-
	Least cost Distance from village	599.81 (415.62)	0.01	-		-	-	-
	Density of large livestock	0.88 (2.03)	0.01	-		-	-	-
	Density of small livestock	1.68 (3.90)	0.01	-		-	-	-
	Livestock Biomass Density	231.23 (471.46)	0.01	-		-	-	-
**Encounter rate at activity centre**	Intercept	NA	NA	-2.55 (95%CI: -3.15–1.94)	-2.51 (-3.12 - -1.91)	-2.12 (-2.51–1.73)	-2.11 (-2.49 - -1.73)	2.49
	Two cameras	Category	0.96	0.56 (95%CI: 0.12–0.99)	0.56 (0.13–1.0)	0.73 (0.31–1.14)	0.73 (0.31–1.14)	
	Gully bed	Category	0.62	0.44 (95%CI: -0.2–1.07)	0.42 (-0.21–1.05)	-	-	0.63 (0.14–1.13)
	Ridgelines	Category	0.62	0.74 (95%CI: 0.11–1.39)	0.71 (0.07–1.35)	-	-	0.97 (0.45–1.49)
**Ranging parameter**	Intercept	NA	0.62	8.75 (95%CI: 8.63–8.88)	8.75 (8.63–8.88)	8.76 (8.63–8.88)	8.76 (8.63–8.88)	8.78 (8.68–8.89)
**Resistance/Conductance parameter**	None	NA	0.0	-	-	-	-	-
	Altitude	4775 (509)	1.0	0.36 (95%CI: 0.21–0.51)	0.39 (0.22–0.55)	0.37 (0.22–0.52)	0.40 (0.27–0.52)	0.38

The model averaged density surface suggests hat only 14% of the entire area of integration had estimated snow leopard density greater than 1 animal per 100 km^2^ from the top model, while 64% had an estimated density lower than 0.25 animals per 100 km^2^.

## Discussion

Our study established the first baseline estimate of the population and density of the snow leopard in Spiti Valley, an important snow leopard habitat in India that has been identified by the Indian Government as a priority landscape under the Global Snow Leopard and Ecosystem Protection Program [38]. In our study area, a combination of community-based conservation efforts over the years, peoples’ religious beliefs, and law enforcement have led to a substantial reduction of retaliatory killing of snow leopards and hunting of ungulates [39]. The estimated snow leopard density in our study area was lower than that from studies conducted in several other smaller study areas [19, 40–42], but there was considerable spatial variation in density in our study area. Our results support the possibility that density estimates from several earlier studies might be positively biased because of small study areas (< 400 km^2^) [19, 43, 44] that were located in high-density parts of respective landscapes [7].

All the top models in our study indicated that conductance is greater at higher altitudes. Ecologically, this can be translated as snow leopards tending to move greater distances at higher altitudes, matching natural history observations that suggest snow leopards move along ridgelines [45–47].

Our top model showed that the variation in snow leopard density was largely associated with variation in wild prey density. It appears, therefore that in multiple-use areas where the killing of snow leopards is not a serious threat, the variation in the abundance of wild prey is the primary determinant of spatial variation in snow leopard density. Models that included ruggedness in addition to wild prey density were a close second, conforming to their preference to certain habitat characteristics [48–50]. Other variables including distance from settlements, livestock biomass, density of large and small livestock, and altitude above mean sea level did not have any noteworthy effect on the snow leopard density within the study area. This is broadly in line with the conclusions of Suryawanshi et al. [29], who have shown that snow leopard abundance is primarily determined by the abundance of wild prey, and not by the abundance of livestock. Snow leopard habitat use and wild prey densities are reported to be lower in areas with high livestock density [51].

Human settlements and associated anthropogenic pressures are considered to negatively influence carnivore habitat use [52, 53]. In the case of snow leopards, studies report conflicting results. For instance, while one study found human settlements to exert a negative influence on snow leopard habitat use [51], other studies reported no such effect [15, 54]. In our study area, human density was low (<2 per km^2^), and livestock grazing the major anthropogenic activity.

**Fig 3:**
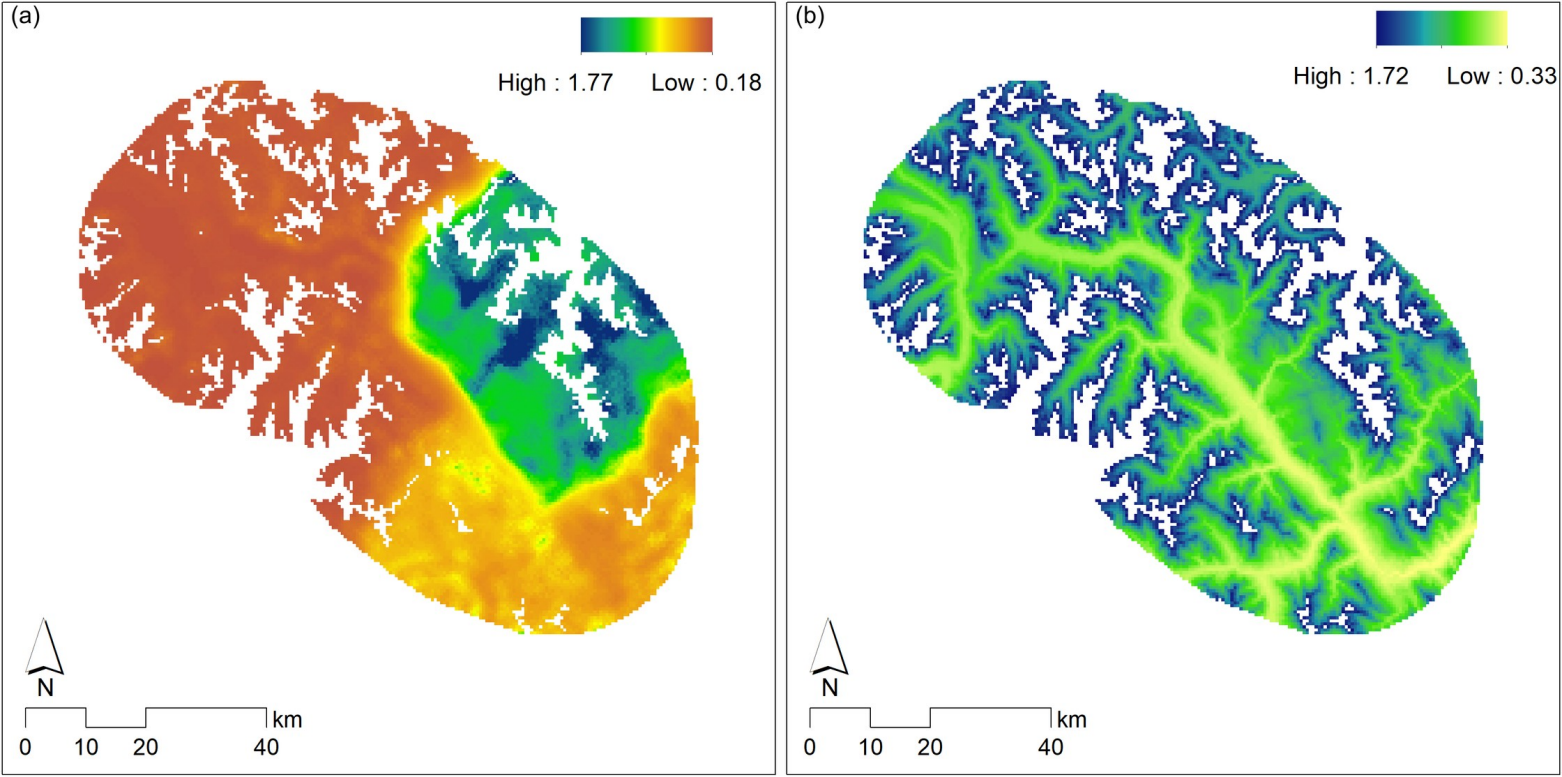
Maps of estimated density and conductance in the Spiti valley based on the model-averaged SCR model using the top three models. (a) Snow leopard density, (b) log of conductance in the habitat for snow leopard movement. The coloured region shows the area of integration. * The data depicted the maps were developed by the authors using models described in the manuscript.

Mishra [55] provided a conceptual framework for a land-sharing approach for wildlife conservation in snow leopard landscapes, that advocates maintaining a matrix of ‘core’ (no grazing or human use) and ‘buffer’ landscape units (grazing and other sustainable human use activities) maintained with community support. Our results suggest that this would be particularly useful in the south-east and north-west parts of Spiti Valley that have low snow leopard density (Fig 3). There is evidence that the creation of such ‘core’ landscape units with community support can lead to the recovery of wild prey, and therefore, of snow leopards [39]. Such efforts require building long term partnerships with local communities by co-opting them in conservation efforts [56].

Our findings suggest that maintaining pockets of high-density wild prey populations can immensely facilitate snow leopard conservation in multiple-use landscapes. They also hint at the possible redundancy of human disturbance and livestock densities for snow leopards in the presence of successful long-term community-based conservation programs. We suggest that the land-sharing approach to snow leopard conservation can be strengthened considerably in snow leopard landscapes of Asia by creating core landscape units that can facilitate the recovery of ungulate populations, while minimizing negative interactions with humans through proactive engagement with local communities.
